# Long-term survival of a patient with metachronous rectal metastasis from primary cecal cancer who underwent repetitive resection and chemotherapy: a case report

**DOI:** 10.1186/1477-7819-12-107

**Published:** 2014-04-23

**Authors:** Jiro Shimazaki, Takeshi Nakachi, Takanobu Tabuchi, Hideyuki Ubukata, Takafumi Tabuchi

**Affiliations:** 1 Department of Gastrointestinal Surgery, Ibaraki Medical Center, Tokyo Medical University, 3-20-1 Chuo Ami, Inashiki, Ibaraki 300-0395, Japan

**Keywords:** Colorectal cancer, Metastasis, Chemotherapy

## Abstract

There are few reported cases of colorectal metastasis from cancers of other organs, particularly other segments of the colon. Here we describe the long-term survival of a 68-year-old male patient with metachronous rectal metastasis from cecal cancer who underwent repetitive resection and chemotherapy. The patient underwent ileocecal resection and hepatectomy for cecal cancer with liver metastasis (T3, N1a, M1a, Stage IVA) in 2006. The patient subsequently underwent splenectomy for splenic metastasis in 2007. In August 2008, barium enema revealed compression of the rectal wall, and abdominal computed tomography (CT) detected a mass along the rectum extending into the pelvis. Rectal metastasis from cecal cancer was suspected and Hartmann’s operation with bilateral seminal vesicle dissection was performed. Histological examination of the excised tumor revealed moderately differentiated adenocarcinoma formed in the muscularis propria of the rectum and infiltrating the connective tissue between the seminal vesicle and rectum. However, no tumor was detected in the rectal mucosa or submucosa. These histological findings supported the diagnosis of rectal metastasis from cecal cancer. The patient has been monitored at our clinic for 60 months after surgical removal of the rectal metastasis. The findings from this case should alert oncologists to the potential danger of rectal metastasis from primary colon cancer and the benefits of timely complete resection in terms of improved patient outcomes.

## Background

Timely detection and improved treatment of metastatic disease have dramatically increased the long-term survival of many cancer patients. Although colorectal cancer is one of the most common neoplasias, it rarely arises as metastasis from cancer in other segments of the colon. There is no consensus regarding the most effective treatment strategy for such cases. Here we report the long-term survival of a 68-year-old male patient with metachronous rectal metastasis from cecal cancer who underwent repetitive resection and chemotherapy.

## Case presentation

A 68-year-old male patient presented at the Department of Gastrointestinal Surgery (Ibaraki Medical Center, Ami, Japan) with progressive abdominal distention and narrowing of the feces for 2 months. The patient’s medical history revealed that he had undergone ileocecal resection for cecal cancer with solitary liver metastasis in September 2006. Light microscopy of the resected specimen revealed moderately differentiated adenocarcinoma infiltrating the deep layers through the muscularis propria of the cecum and metastases in 1 of 14 lymph nodes. The tumor was diagnosed as stage IVA (T3, N1a, M1a) according to the International Union Against Cancer tumor, node, and metastasis (TNM) classification (7th edition) [1]. After oral tegafur/uracil and oral leucovorin combination chemotherapy for 3 months, the solitary liver metastasis was resected by partial hepatectomy in December 2006. The patient subsequently underwent splenectomy for splenic metastasis in 2007.

**Figure 1:**
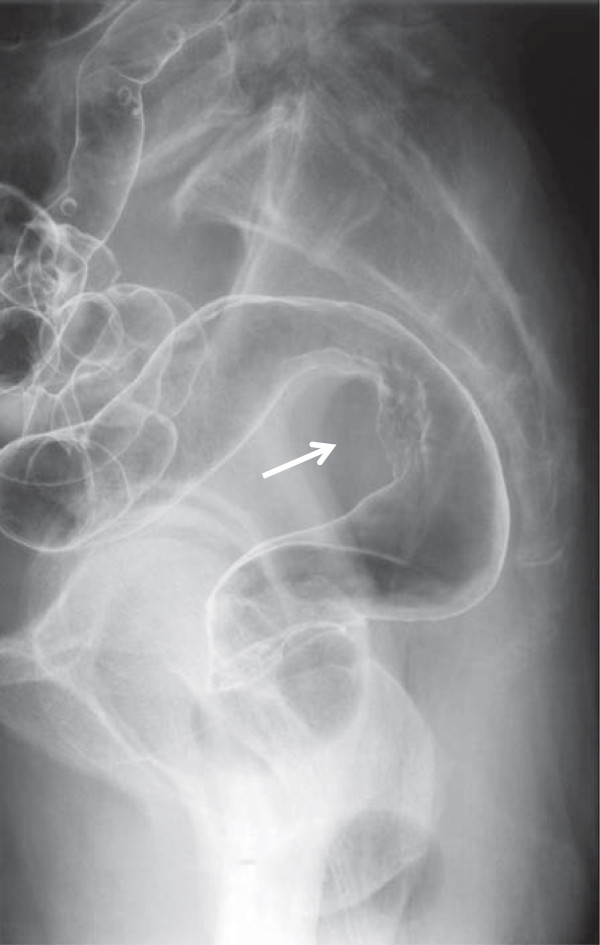
Barium enema reveals compression of the rectal wall and a partially uneven mucosal surface (arrow).

**Figure 2:**
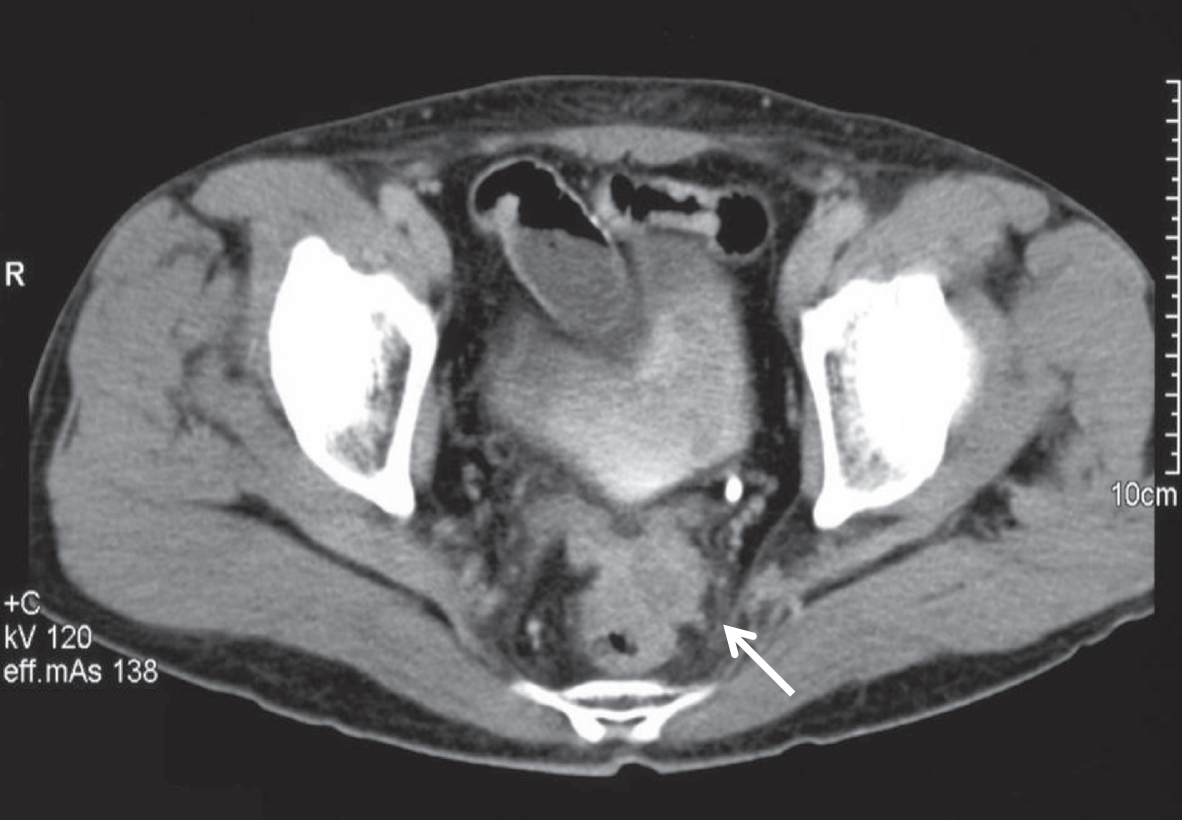
Abdominal computed tomography (CT) reveals a mass measuring 40 mm in diameter along the rectal wall and extending into the pelvis (arrow).

The findings of subsequent physical examination were unremarkable except for slight pallor in the palpebral conjunctiva. Hematological investigation revealed anemia (hemoglobin, 10.6 g/dl; hematocrit, 31.4%), while other laboratory test results and serum levels of carcinoembryonic antigen and carbohydrate antigen 19-9 were within normal limits. Barium enema revealed compression of the rectal wall with a partially uneven mucosal surface (Figure 1), and colonoscopy revealed slight mucosal erythema at the rectum. No tumor was detected in mucosal biopsy specimens; however, abdominal computed tomography (CT) revealed a pelvic mass measuring 40 mm in diameter along the rectum (Figure 2). We suspected rectal metastasis from cecal cancer and performed Hartmann’s operation with bilateral seminal vesicle dissection in August 2008.

**Figure 3:**
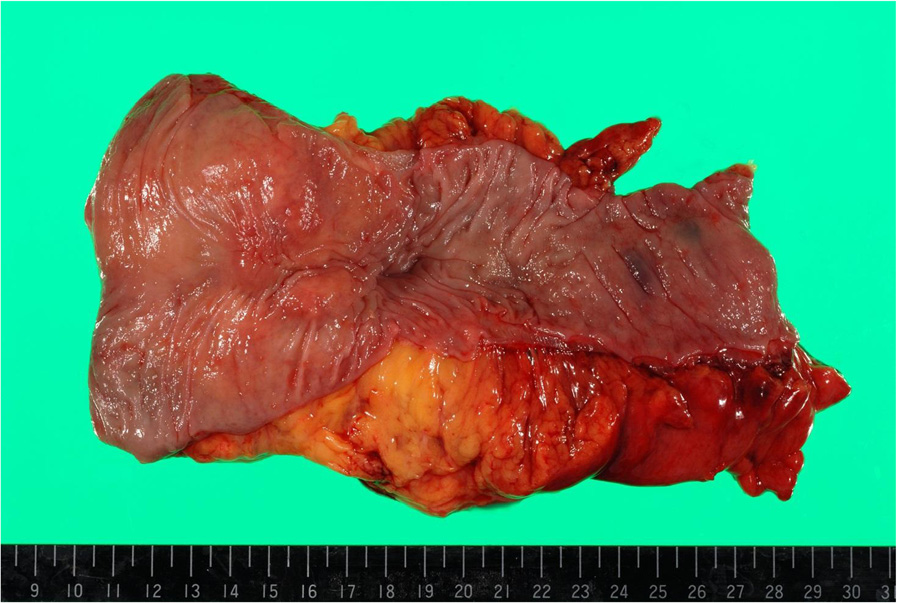
**Macroscopic findings from the resected specimen.** The tumor is submucosal with an uneven mucosa and measures 5.5 × 4.0 × 3.5 cm.

**Figure 4:**
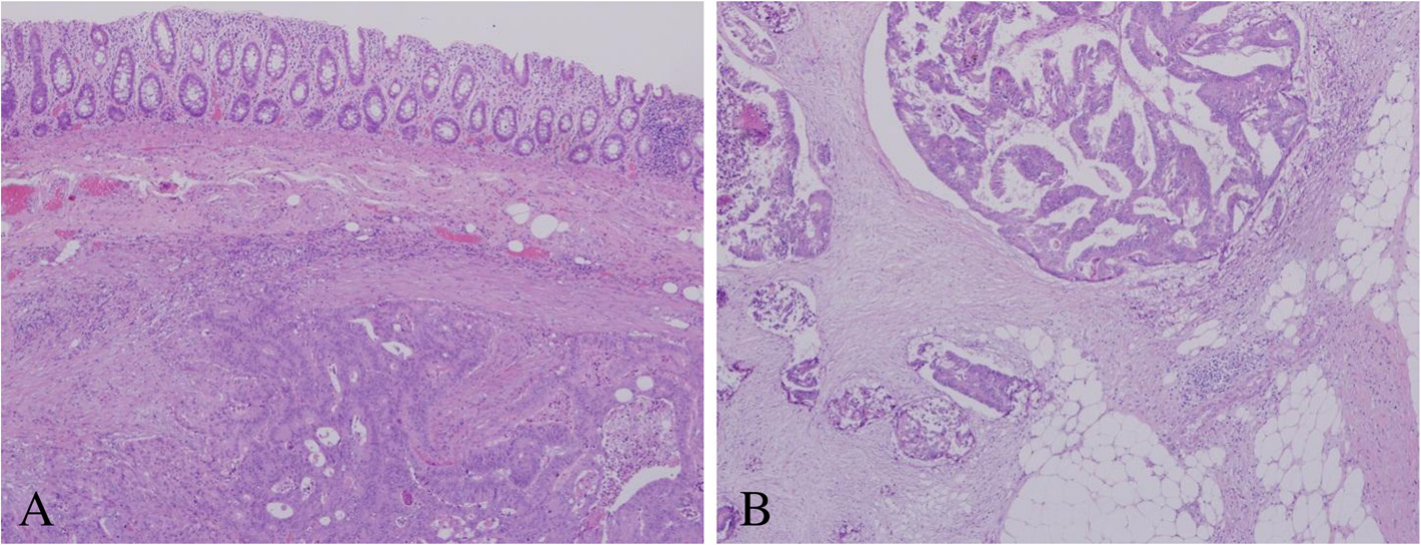
**Histological analysis of the resected specimen. (A)** Moderately differentiated adenocarcinoma formed in the propria muscularis of the rectum (H & E, 40×). **(B)** Tumor cells infiltrating the connective tissue between the seminal vesicle and rectum (H & E, 200×). H & E, hematoxylin and eosin.

The mass had penetrated the anterior wall of the rectum below the peritoneal reflection. There were no intraoperative findings of disseminated tumor in the abdominal cavity, findings inconsistent with disseminated peritoneal metastasis. The resected tumor measured 5.5 × 4.0 × 3.5 cm and had an uneven surface (Figure 3). Sections of the tumor revealed solid gray, yellowish myxoid areas. Histological examination of the excised tumor showed moderately differentiated adenocarcinoma formed in the muscularis propria of the rectum and infiltrating the connective tissue between the seminal vesicle and rectum (Figure 4A,B). However, no tumor was apparent in the rectal mucosa or submucosa. These findings supported the diagnosis of rectal metastasis from the cecal cancer treated in September 2006.

Abdominal CT performed 6 months later in April 2009 revealed liver metastases, and the patient was treated with fluorouracil, leucovorin, and oxaliplatin + bevacizumab combination therapy. The patient exhibited complete initial clinical response; however, follow-up abdominal CT revealed growth of the liver metastases. The patient refused continued chemotherapy because of peripheral neuropathy caused by oxaliplatin and subsequently underwent partial hepatectomy in October 2011, pneumonectomy for lung metastasis in April 2012, and partial hepatectomy for liver metastasis in September 2012. Following the surgery to remove the rectal metastasis, the patient has been monitored at our outpatient clinic for 60 months.

## Discussion

Cases of colorectal metastasis from cancers of other organs have occasionally been reported [2-5], but colorectal metastasis from other segments of the colon is extremely rare. Nakamura *et al*. [6] and Kalaitzis *et al*. [7] reported single cases of rectal metastasis from cancer of the sigmoid colon, and this is the first report, as per our knowledge, of rectal metastasis from cecal cancer. In our patient, metachronous rectal metastasis originated from cecal cancer resected 2 years back. We suspect a hematogenous metastatic pathway for the following reasons. First, the patient has a history of liver and splenic metastasis from the cecal cancer, indicating systemic hematogenous metastasis. Second, rectal metastasis was present below the peritoneal reflection, and there was no disseminated tumor in the abdominal cavity, ruling out disseminated peritoneal metastasis. Third, tumor cells proliferated mainly in the rectal wall, ruling out lymph node metastasis.

The survival of patients with metastatic colorectal cancer has improved with combined therapies, including chemotherapy [8]. However, there is no consensus strategy for the diagnosis and treatment of metastasis from other sites of primary colorectal cancer. Inada *et al*. [4] reported that immunohistochemical staining is effective for diagnosis. In the present case, however, preoperative histological examinations with colonoscopy did not detect rectal metastasis because the tumor was entirely submucosal. We selected surgery to treat the rectal tumor because preoperative histological examinations were inconclusive and definitive histological diagnosis was required to establish treatment. It remains debatable whether resection of metastases from other sites of primary colorectal cancer can improve outcome because no similar report has been published. However, the Japanese guidelines for the treatment of colorectal cancer recommend surgery for metastatic lesions if the primary colorectal lesion and metastases are completely resectable and the performance status of the patient is acceptable [9]. In the present case, rectal metastasis was the only tumor detectable by preoperative examination; furthermore, the performance status of the patient was good. Therefore, resection of the rectal tumor may have contributed to successful outcome in the present case.

The patient’s tumor metastasized to the liver and lung after Hartmann’s operation. Fluorouracil, leucovorin, and irinotecan + bevacizumab combination therapy was an option for second-line therapy against the metastases [10,11]; however, the patient refused additional chemotherapy because of intolerable side effects and opted for surgery instead. The 5-year survival rates after hepatectomy for liver metastasis and pneumonectomy for lung metastasis from colorectal cancer are 35 to 58% and 30 to 68%, respectively [12-15]. Moreover, the overall survival of patients with completely resectable lung metastases is better than that of patients with unresectable lung metastases [15,16]. The patient has been monitored for 5 years after Hartmann’s operation, and repetitive resection and chemotherapy for the metastases appear to have contributed to long-term survival.

## Conclusions

Colorectal metastasis from other sites of primary colon cancer is extremely rare, and complete resection of these metastatic lesions may significantly improve outcome. The findings from this case should alert oncologists to the potential danger of rectal metastasis from primary colon cancer and the benefits of timely complete resection.

## Consent

Written informed consent was obtained from the patient for publication of this case report and any accompanying images. A copy of the written consent is available for review by the Editor-in-Chief of this journal.

## Abbreviations

CT: Computed tomography; H & E: Hematoxylin and eosin; TNM: Tumor node, and metastasis.

## Competing interests

The authors declare no competing interests or competing financial interests with respect to the authorship and/or publication of this article.

## Authors’ contributions

JS wrote the manuscript. TN and TT performed surgery. HU and TT drafted the manuscript. All authors read and approved the final manuscript.
